# Influence of Genotype and Environment on Arabinoxylan Content and Structural Characteristics in Grain and Flour of Organically Grown Winter Rye

**DOI:** 10.3390/molecules31193376

**Published:** 2026-09-23

**Authors:** Sylwia Stępniewska, Anna Fraś, Magdalena Wiśniewska, Krzysztof Buksa, Grażyna Cacak-Pietrzak, Marcin Studnicki, Dariusz Dziki

**Affiliations:** 1 Department of Food Technology and Assessment, Institute of Food Sciences, Warsaw University of Life Sciences (WULS), Nowoursynowska 159C Street, 02-787 Warsaw, Poland; grazyna_cacak_pietrzak@sggw.edu.pl; 2 Department of Grain Processing and Bakery, Prof. Wacław Dąbrowski Institute of Agricultural and Food Biotechnology—State Research Institute, Rakowiecka 36 Street, 02-532 Warsaw, Poland; 3 Plant Breeding and Acclimatization Institute—National Research Institute, Radzików, 05-870 Błonie, Poland; a.fras@ihar.edu.pl (A.F.); m.wisniewska@ihar.edu.pl (M.W.); 4 Department of Carbohydrate Technology and Cereal Processing, University of Agriculture in Krakow, Balicka 122, 30-149 Krakow, Poland; krzysztof.buksa@urk.edu.pl; 5 Department of Biometry, Institute of Agriculture, Warsaw University of Life Sciences, Nowoursynowska 159 Street, 02-776 Warsaw, Poland; marcin_studnicki@sggw.edu.pl; 6 Department of Thermal Technology, Lublin University of Life Sciences, Głęboka 31 Street, 20-612 Lublin, Poland

**Keywords:** arabinoxylans, ferulic acid, molar mass, organic production, rye

## Abstract

The aim of the study was to determine the effects of environmental factors (harvest year and growing location), genotype, and their interactions on the content, molar mass, degree of arabinose substitution, and ferulic acid content of arabinoxylans (AXs) in organically grown rye grain and the corresponding low-extraction rye flours. The experimental material consisted of grain from five population cultivars and two hybrid rye cultivars. Genotype, harvest year, growing location, and their interactions affected most of the analyzed arabinoxylan characteristics, although the significance and magnitude of these effects depended on the arabinoxylan fraction and material analyzed. Rye grain was characterized by a higher total AXs content but a lower proportion of the water-extractable fraction compared with the flour. The molar mass of water-extractable AXs ranged from 1.53 × 10^6^ to 2.79 × 10^6^ g/mol in rye grain and was lower in flour (1.27 × 10^6^–2.35 × 10^6^ g/mol), indicating that the high-molar-mass AXs are predominantly localized in the outer layers of the grain. For the first time, it was observed that in the whole grain high-molar-mass AX molecules are less substituted with ferulic acid than AXs of low molar mass. This association may contribute to a better understanding of the structural characteristics of rye cell wall polysaccharides and could provide useful preliminary information for rye breeding and the development of rye-based foods with desirable technological and nutritional properties.

## 1. Introduction

Rye (*Secale cereale* L.) is a bread cereal cultivated mainly in Northern, Eastern, and Central Europe. Due to its low soil requirements and exceptional winter hardiness, it can be grown on light soils and in regions with harsh climatic conditions. Importantly, rye can be cultivated without the use of herbicides, which facilitates obtaining yields from a more environmentally friendly and sustainable production system [1]. Organic rye cultivation avoids the use of synthetic fertilizers and pesticides, which contribute to soil and water contamination and adversely affect beneficial insects and microorganisms. This practice is consistent with the principles of sustainable agriculture, as it reduces environmental degradation and mitigates the impacts of climate change [2]. These characteristics make rye a suitable crop for organic farming systems. However, differences in environmental conditions and cultivation systems may affect the composition and technological properties of rye grain and may interact with cultivar-specific characteristics.

Rye grain is a rich source of dietary fiber, with arabinoxylans (AXs) being the predominant compounds. These non-starch polysaccharides play a significant role in determining the physical and chemical properties of rye grain [3,4]. Arabinoxylans consist of a linear backbone of β-D-xylopyranosyl units substituted with arabinofuranosyl side chains [5,6]. Some arabinose residues in AX are ester-linked to ferulic acid, which contributes to their bioactive properties [7]. The degree of branching and the presence of ferulic acid attached to arabinose residues influence the functional and physiological properties of AXs [3,8]. Arabinoxylans provide numerous health benefits, affecting colon health, lipid metabolism, and glycemic control [9,10]. Water-extractable AXs exhibit prebiotic properties by promoting the growth of beneficial gut bacteria. They pass undigested through the small intestine and reach the colon, where they are fermented by intestinal microbiota, producing beneficial short-chain fatty acids (SCFAs), such as acetate, butyrate, and propionate. These SCFAs support colonic health and improve bowel function [11]. Arabinoxylans have also been reported to exhibit immunomodulatory, antioxidant, and anticancer properties and to influence metabolic and gastrointestinal functions [12,13,14].

The total arabinoxylan (T-AX) content in rye grain typically ranges from 8 to 12% of dry matter, with the two main fractions being water-extractable arabinoxylans (S-AX) and water-unextractable arabinoxylans (I-AX) [15]. S-AX constitute a relatively small proportion of total AX and are loosely associated with the surface of the cell wall, whereas the predominant insoluble fraction remains tightly bound to other cell wall components, such as cellulose, lignin, and proteins [6]. Arabinoxylans exert a significant influence on the rheological properties of dough and the quality of bakery products. S-AX exhibit beneficial technological effects, as they increase the water-binding capacity of dough, improve its stability during mixing and fermentation, and contribute to enhanced bread volume. Owing to these properties, dough becomes more elastic and more resistant to mechanical stress during processing [16]. In contrast, I-AX often exhibit adverse technological effects. This fraction is strongly associated with the cell wall matrix and may disrupt the porous structure of fermenting dough by destabilizing gas cells, resulting in reduced bread volume and deterioration of the texture and sensory attributes of the final product. Therefore, a high content of I-AX in flour may negatively affect bread quality, representing an important technological challenge in bakery production. Consequently, there are distinct differences in the content and proportion of S-AX and I-AX among various milling fractions. Low-extraction flours contain higher levels of soluble AX, whereas high-extraction flours are characterized by a greater content of the insoluble fraction [1].

Therefore, comparison of whole grain with low-extraction flour is relevant for evaluating differences in AX content and structural characteristics associated with milling. Such a comparison is particularly useful for determining whether differences observed in whole grain are reflected in the corresponding flour used for further cereal processing. Previous studies have focused primarily on rye produced under conventional cultivation, leaving a significant knowledge gap concerning the effects of genotype, environmental conditions, and agronomic practices in organic systems on the quantitative and qualitative profiles of AXs. In particular, limited information is available on the combined effects of genotype, harvest year, and growing location on AXs characteristics in organically grown rye. Therefore, the present study was undertaken to evaluate the content and structural characteristics of arabinoxylans in grains of different rye cultivars, including both population and hybrid varieties, cultivated under organic farming conditions across different locations and growing seasons. This approach enables the assessment of both genotypic and environmental variability, which may significantly influence the concentration and properties of AXs in rye grain and flour. From a nutritional perspective, these investigations are particularly important. A substantial proportion of the population fails to achieve the recommended daily intake of dietary fibre, as confirmed by recent epidemiological studies [17,18]. The identification of genotypes and environmental conditions associated with specific AXs characteristics may therefore be relevant to rye breeding and cereal processing. The findings of the present study may contribute to a better understanding of the variability of AXs in organically grown rye and its relevance to cereal processing.

The aim of the study was to determine the effects of environmental factors (harvest year and growing location), genotype, and their interactions on AXs content, molar mass, degree of arabinose substitution, and ferulic acid content in grain from selected hybrid and population rye cultivars grown under organic farming conditions and in the corresponding rye flours.

## 2. Results and Discussion

### 2.1. Arabinoxylan Content in Tested Rye Grain Samples

Table 1 presents the contents of individual arabinoxylan fractions and the share of water-extractable arabinoxylan in total arabinoxylans content in the grain of the investigated rye cultivars grown under organic farming conditions.

The total arabinoxylans (T-AX) content of the grain of the studied rye cultivars was within the typical range reported for rye, varying from 7.18 to 9.12% dry matter (d.m.) [17,19]. For comparison, grain of four rye cultivars cultivated under intensive and integrated production systems and examined by Buksa et al. [15] contained T-AX in the range of 8.40–10.40% d.m. In an earlier study by the same authors, three population rye cultivars grown under conventional farming conditions and harvested between 2004 and 2006 exhibited a wider range of T-AX contents, from 7.1 to 12.2% d.m. [20]. Based on the results presented in Table 1, the harvest year had a significant effect on T-AX content. Grain harvested in 2020 had a higher T-AX content than grain harvested in 2019, while a smaller but significant location effect was also observed. Cultivar differences were also evident, with Tur and Dańkowskie Skand showing the lowest and highest mean T-AX contents, respectively (Table 1). The influence of genotype and environment on T-AX accumulation remains an important issue, particularly in organic rye production, where environmental variability may contribute substantially to differences in grain composition. Finnie et al. [21] reported that T-AX content was determined to a greater extent by genetic factors than by environmental conditions. In contrast, Tremmel-Bede et al. [22] found no significant effect of genotype on T-AX content. Li et al. [23], however, demonstrated a predominant influence of environmental conditions on T-AX accumulation, suggesting that plant responses may strongly depend on cultivation conditions and their interaction with genetic background. Studies conducted by Rakszegi et al. [24] revealed a significant influence of abiotic stress, particularly heat and water stress, on T-AX content in wheat grain. The highest T-AX concentrations were observed in cultivars exposed simultaneously to heat and drought stress. Although all stress treatments generally increased T-AX levels, drought stress alone resulted in decreased arabinoxylan content in drought-tolerant cultivars. These findings indicate substantial variation in the metabolic responses of wheat genotypes to environmental stress, depending on their susceptibility or tolerance to specific stress factors. Similarly, Korge et al. [9], who investigated winter wheat and spring barley cultivated under conventional and organic farming systems in Estonia during 2014–2021, demonstrated a significant effect of weather conditions on grain arabinoxylan content, with particular emphasis on temperature during the grain-filling period. Elevated temperatures during this developmental stage promoted arabinoxylan accumulation. Furthermore, organically grown grain contained higher levels of arabinoxylans than conventionally grown grain, although this difference was statistically significant only in winter wheat harvested during seasons characterized by lower temperatures during grain filling. Their analysis also indicated that cereal species exerted a stronger influence on arabinoxylan content than cultivar, with wheat containing higher AXs levels than barley.

From the perspective of organic rye production, the observed variation in AXs content is particularly relevant because the environmental conditions under which organically grown crops are produced may substantially affect grain composition. The significant effects of harvest year and cultivation location observed in the present study indicate that environmental variability should be considered when evaluating the AXs profile of organically produced rye. At the same time, the significant contribution of cultivar and the observed genotype × environment interactions suggest that the choice of cultivar may provide an important means of managing this variability. Thus, cultivars differ not only in their average AXs content but also in the extent to which their AXs characteristics respond to changing environmental conditions. From a practical perspective, the identification of cultivars showing a relatively stable and desirable AXs profile across organic production environments may therefore be important for obtaining rye grain with more predictable technological and functional properties.

The content of water-extractable arabinoxylan (S-AX) in the grain of the studied rye cultivars ranged from 2.08 to 2.76% d.m. (Table 1). Ismagilov et al. [25] reported S-AX contents in rye grain within a slightly narrower range (2.54–2.75% d.m.). In contrast, Kozlova et al. [26] demonstrated considerably greater variability in S-AX content in the grain of six rye cultivars originating from Russia (1.22–4.01% d.m.). The S-AX content in wheat grain analyzed by Li et al. [23] was substantially lower than that observed in rye in the present study and did not exceed 1% d.m. The authors further demonstrated that environmental factors exerted a stronger influence on S-AX content than genetic factors, findings consistent with those of the present study. Available literature indicates that S-AX content in rye grain is significantly affected by total precipitation and mean temperature during the period from heading to harvest. Shewry et al. [27] reported a positive correlation with precipitation and a negative correlation with mean air temperature during this developmental stage. These findings suggest that cool and moist conditions favor the accumulation of S-AX in cereal grains. However, these relationships may be modified by increased xylanase activity under conditions of high moisture and low temperature, which may enhance arabinoxylan degradation [28]. In the present study, S-AX content was higher in grain harvested in 2019 than in 2020, while the Osiny site also showed higher content of S-AX than Grabów (Table 1). The differences between years coincided with substantial differences in precipitation during May–July. The present study also demonstrated a genetic effect on S-AX content. Two population cultivars, namely Dańkowskie Granat and Dańkowskie Skand, were characterized by significantly higher S-AX contents than the remaining studied cultivars (Table 1).

The content of water-unextractable arabinoxylan (I-AX) in the grain of the studied rye cultivars grown under organic farming conditions ranged from 4.56 to 6.50% d.m. (Table 1). According to Finnie et al. [21], I-AX content is primarily determined by environmental factors, such as weather conditions and agronomic practices during the growing season. In the present study, both harvest year and cultivar had a significant effect on the formation of this arabinoxylan fraction. I-AX content was higher in grain harvested in 2020 than in 2019, whereas no significant effect of cultivation location was observed (Table 1). The results suggest that total precipitation during May-July may play an important role in determining I-AX content. Although only minor differences in precipitation sums were observed between the study locations within a given growing season, consideration of harvest year revealed clear differences in rainfall levels during this period, which may explain the observed variability in I-AX content. Regarding the genetic factor, the hybrid cultivar Tur exhibited the lowest I-AX content among the studied cultivars.

In addition to the content of individual arabinoxylan fractions in rye grain, the proportion of water-extractable arabinoxylan in total arabinoxylans (%S-AX) is also an important parameter. In the present study, %S-AX ranged from 24 to 37% and was comparable to values reported for rye grain investigated by Hansen et al. [29,30], where %S-AX ranged from 26 to 39%. In contrast, studies by Ponomareva et al. [31] showed substantially lower %S-AX levels in winter rye cultivars, not exceeding 15%. According to Khurshid et al. [32], cultivar was the main factor influencing %S-AX, with a significant effect also observed for the interaction between cultivar and growing year. In the present study, however, the dominant factor affecting %S-AX was the harvest year, whereas the effect of genotype was considerably weaker. Rye grain harvested in 2019 exhibited a significantly higher %S-AX compared with grain harvested in 2020 (Table 1). As precipitation during May–July in 2020 was substantially higher in both cultivation sites than in 2019, the observed lower %S-AX in 2020 may be associated with the higher precipitation during the heading-to-harvest period. This observation is consistent with the findings of Khurshid et al. [32], who reported a moderate negative correlation between average precipitation during the heading-to-harvest period and %S-AX. In the present study, grain produced at the Osiny location exhibited a significantly higher %S-AX than grain from the Grabów site. Regarding the genetic effect, the hybrid cultivar Tur and the two population cultivars, Dańkowskie Granat and Dańkowskie Skand, showed significantly higher %S-AX values than the remaining studied cultivars.

These findings are also relevant to organic rye production, where year-to-year variation in weather conditions may contribute substantially to the variability of grain quality. In the present study, the contrasting precipitation conditions between the 2019 and 2020 growing seasons were accompanied by significant differences in both S-AX content and the proportion of S-AX in total AX. Although the present experimental design does not allow the effect of the organic production system itself to be separated from environmental effects, the results indicate that weather variability within organic production environments can contribute to substantial changes in the AX content of rye grain. Consequently, assessment of cultivar performance under organic conditions should consider not only the mean AX concentrations but also the balance between water-extractable and water-unextractable AX fractions under growing conditions.

### 2.2. Arabinoxylan Content in Tested Rye Flour Samples

Table 2 presents the results of total, water-extractable, and water-unextractable arabinoxylan contents, as well as the proportion of water-extractable arabinoxylan in total arabinoxylans, in the studied rye flour samples.

The total arabinoxylans content in flour (T-AX_F_) obtained from the grain of the investigated rye cultivars ranged widely from 2.87 to 4.17% d.m. This level was considerably lower than the T-AX_F_ content reported for low-extraction rye flours produced in industrial milling plants studied by Stępniewska et al. [33], where T-AX_F_ exceeded 4.57% d.m. and reached an average of 5.92% d.m. These differences may result from variations in milling technology and the use of grain originating from different agricultural production systems. In the previous study by Stępniewska et al. [33], the grain was derived from commercial agricultural production, and different milling technologies were applied in each industrial mill. In contrast, in the present study, milling of all rye cultivars was performed under laboratory conditions using an identical milling procedure. In the present study, T-AX_F_ was significantly affected by both harvest year and cultivation location, whereas the magnitude of cultivar differences was smaller (Table 2). This indicates that environmental variation may contribute substantially to the AX content of the resulting flour. In contrast, previous studies by Stępniewska [33] did not report significant variation in T-AX_F_ content in rye flour produced in industrial mills across different production years. In the present study, flour obtained from grain cultivated at the Grabów site exhibited significantly higher T-AX_F_ content (mean 3.58% d.m.) than flour produced from grain grown at the Osiny site (mean 3.31% d.m.). Interestingly, flour from Osiny grain, despite originating from grain with significantly higher T-AX content, showed a lower final T-AX_F_ level compared with flour derived from Grabów grain. This is likely related to the milling methodology, particularly the standardization of flour extraction rate to 55%. In the case of grain from Osiny, a smaller proportion of bran-rich flour (rich in arabinoxylans) was incorporated into the final flour blend to achieve the required extraction rate, which resulted in a lower final T-AX_F_ content. Furthermore, the present study demonstrated a significant effect of cultivar on T-AX_F_ content. However, both population and hybrid cultivars showed a comparable range of T-AX_F_ values (3.32–3.53% d.m. and 3.40–3.59% d.m., respectively). The highest T-AX_F_ contents were observed in flour obtained from the hybrid cultivar Tur and the population cultivar Dańkowskie Hadron (mean 3.53 and 3.59% d.m., respectively), whereas the lowest T-AX_F_ level was found in flour derived from the hybrid cultivar KWS Dolaro (mean 3.32% d.m.).

The content of water-extractable arabinoxylan (S-AX_F_) in rye flours obtained from milling grain of the studied cultivars grown under organic farming conditions ranged from 1.74 to 2.44% dry matter (d.m.). The S-AX_F_ content was higher than that of the water-unextractable arabinoxylan fraction. A similar relationship was reported by Cyran and Cygankiewicz [34], who investigated low-extraction rye flours obtained from grain originating from different regions of Poland and harvested between 1996 and 1998. In those studies, the S-AX_F_ content in rye flour was higher than that observed in the present work and ranged from 2.10 to 3.40% d.m. The observed differences may be attributed to differences in flour extraction rates. In the present study, rye flours with a fixed extraction rate of 55% were analyzed, whereas Cyran and Cygankiewicz [34] examined flours with varying extraction levels (44–68%), which may have contributed to greater variability and higher levels of water-extractable arabinoxylan. For comparison, in earlier studies by Stępniewska et al. [33], low-extraction flours produced in industrial milling plants also showed higher S-AX_F_ contents than those observed in the present study (mean 3.53% d.m.). This is likely related either to higher S-AX content in the grain used for milling or to a greater proportion of flour fractions derived from the outer endosperm layers, which are rich in water-extractable arabinoxylan [1]. Statistical analysis revealed a significant effect of both harvest year and cultivar on S-AX_F_ content. Flour obtained from grain harvested in 2019 had a significantly higher S-AX_F_ content than that produced from grain harvested in 2020 (Table 2). It was also found that flour obtained from hybrid rye cultivars was less favorable in terms of S-AX_F_ content compared with flour derived from population cultivars. In contrast, different results were reported by Cyran and Dynkowska [35], who observed significantly higher T-AX_F_ contents in low-extraction flours obtained from hybrid rye cultivars (2.21–2.74% d.m.) than in those derived from population cultivars (1.92–2.08% d.m.). S-AX_F_ content differed among cultivars, with the highest mean values observed for the population cultivars Dańkowskie Hadron and Dańkowskie Granat, whereas the lowest value was found for the hybrid cultivar KWS Dolaro (Table 2). In contrast, cultivation location had no statistically significant effect on S-AX_F_ content, with comparable values observed for flours obtained from grain cultivated at Osiny and Grabów. The content of water-unextractable arabinoxylan (I-AX_F_) in the studied rye flours was markedly lower than that observed in the grain from which these flours were obtained. I-AX_F_ content ranged from 0.85 to 2.16% dry matter (d.m.). A wider range of I-AX_F_ contents was reported in low-extraction rye flours produced in industrial mills located in Poland during 2019–2020 and analyzed in earlier studies by Stępniewska et al. [33]. All experimental factors had a significant effect on I-AX_F_ content. Flours obtained from grain harvested in 2019 were characterized by significantly lower I-AX_F_ levels compared with those produced from grain harvested in 2020. Although the I-AX content of grain did not differ significantly between the two cultivation locations, the corresponding flours differed in I-AX_F_ content, with lower values observed for flour from Osiny than for flour from Grabów. This observation may be explained by differences in the distribution of arabinoxylans within specific anatomical parts of the kernel as well as by differences in milling properties of the grain. Insoluble arabinoxylan is predominantly located in the aleurone layer and the pericarp; therefore, at a low flour extraction rate (55%), even minor differences in the incorporation of outer grain fractions into the flour may result in significant variation in I-AX_F_ content in the final product [36]. Statistical analysis revealed that flour obtained from grain of the hybrid cultivar Tur and the population cultivar Dańkowskie Hadron exhibited the highest I-AX_F_ contents (means 1.55 and 1.51% d.m., respectively). In contrast, the lowest I-AX_F_ content was observed in flour produced from the population cultivar Dańkowskie Granat (mean 1.35% d.m.).

The proportion of water-extractable arabinoxylan in total arabinoxylans (%S-AX_F_) in the studied rye flours ranged from 47 to 70%. Considerably lower %S-AX_F_ values were reported in high-extraction rye flours investigated by Stępniewska et al. [37], where %S-AX_F_ ranged from 31 to 48%. The observed differences are mainly attributable to the use of flours with different extraction rates. In the present study, low-extraction flours were analyzed, which are characterized by a higher proportion of particles derived from the endosperm and a lower contribution of the pericarp and seed coat, where the content of water-extractable arabinoxylans is lower [1,5]. In contrast, the study by Stępniewska et al. [37] focused on high-extraction flours, which contain larger amounts of outer grain layers rich mainly in water-unextractable arabinoxylans. As a consequence, the %S-AX_F_ in those flours was markedly lower. Flours obtained from grain harvested in 2019 had significantly higher %S-AX_F_ values than those obtained from grain harvested in 2020 (mean 65% vs. 53%), while flour from Osiny had higher %S-AX_F_ values than those from Grabów (mean 61% vs. 56%). Among the studied cultivars, flour produced from the population cultivar Dańkowskie Granat showed the highest %S-AX_F_, whereas the lowest value was observed for flour obtained from the hybrid cultivar Tur.

From the perspective of organic rye processing, these results indicate that environmental variability during grain production may be reflected in the AXs profile of the resulting flour, although the milling process may modify the extent of this variation. Therefore, the selection of cultivars for organic production should be considered alongside the intended end use and milling process, particularly when a specific AXs profile is desired in low-extraction rye flour.

### 2.3. Structure of Arabinoxylans in Tested Rye Grain Samples

Table 3 presents the structural characteristics of arabinoxylans extracted from rye grain. The physicochemical properties of rye arabinoxylans are largely determined by their heterogeneous structural composition. Four types of substitution patterns can be distinguished along the xylose chain: unsubstituted fragments, and fragments substituted with an arabinose residue at the O-2 position, the O-3 position, or both the O-2 and O-3 positions [38]. Unsubstituted fragments within the AXs chain exhibit a tendency to aggregate, which reduces the polysaccharide’s solubility. Conversely, the soluble arabinoxylan fraction typically contains polymer molecules characterized by a high degree of substitution. On the other hand, highly substituted arabinoxylan chain fragments are less susceptible to enzymatic degradation [39]. Thus, the degree of substitution of the xylan backbone with arabinose residues (A/X ratio) serves as a key indicator of the polysaccharide’s overall degree of branching. Literature reports indicate that the A/X ratio of arabinoxylan chains in rye grain ranges widely, from 0.49 to 0.82 [11]. Meanwhile, the I-AX and S-AX fractions of rye grain are characterized by A/X ratios in the ranges of 0.52–0.68 and 0.34–0.85, respectively [1,11]. In the presented results, the mean A/X ratio for the insoluble arabinoxylan fraction ranged from 0.59 to 0.77, while for S-AX it was 0.59–0.68. Regardless of the year or location of cultivation, the degree of arabinose substitution on the AX chain was slightly lower in S-AX than in I-AX. For both arabinoxylan fractions, a significant genetic influence on the A/X branching degree of the arabinoxylan chain was observed; however, the genetic factor had a stronger influence on the A/X substitution degree in the insoluble arabinoxylan fraction. The KWS Dolaro cultivar exhibited the highest degree of arabinoxylan branching in both the I-AX (0.71) and S-AX (0.65) fractions of the rye grain. Hansen et al. [29] demonstrated a significant effect of genotype on the A/X substitution degree in the water-unextractable AX fraction, and of the cultivation year on the A/X substitution degree in both the soluble and insoluble AXs fractions. The A/X values obtained by these authors fell within the ranges of 0.56–0.66 and 0.64–0.85 for the I-AX and S-AX fractions, respectively.

In order to better characterize the effects of genotype and environment on arabinoxylans in rye grain, water-extractable arabinoxylans were extracted from wholemeal under standardized conditions and their molar mass was determined by size exclusion chromatography (SEC). The use of a refractometric detector in the chromatographic system allowed the determination of the molecular weight distribution of arabinoxylan, while the use of a UV detector operating at a wavelength of 320 nm enabled the detection of ferulic acid molecules attached to arabinoxylan chains depending on their length [15]. Table 3 presents the molar mass of AX molecules and the area under the peak detected at 320 nm. As shown in Table 3, the weight-average molar mass of AXs ranged from 1.53 × 10^6^ to 2.79 × 10^6^ g/mol. This range was similar to that previously reported for water-soluble arabinoxylans extracted from rye wholemeal according to the same procedure [7,15]. However, Dervilly-Pinel et al. [40] reported lower AX molar masses. This difference could be explained by the differences in raw material and especially by using different methods of AX extraction and analysis. Statistical analysis of the data obtained using standardized conditions of sample preparation and analysis (Table 3) revealed that environmental conditions in different years of cultivation had a significant effect on the molar mass of AXs, as the AXs from grain harvested in 2020 had a significantly higher molar mass. Studies on the effect of growing conditions on AX particle size have shown that water availability and rainfall in the year of cultivation may significantly impact the biosynthesis of arabinoxylans [20,41]. The location also influenced the molar mass of AXs in rye grain. According to the results in Table 3, rye grain cultivated in Grabów contained AXs of higher molar mass than grain cultivated in Osiny. Studies conducted so far show that factors such as temperature, humidity, and location affect AXs structure, altering its molar mass [15,20]. Rye cultivar had the most pronounced effect on the molar mass of AXs. It was shown that the Dańkowskie cultivars Granat, Hadron, Skand, and Turkus, particularly Dańkowskie Hadron, as well as Piastowskie produced grain with AXs of significantly higher molar mass than cultivars Tur and KWS Dolaro. The observed strong influence of genotype on molar mass of AX is well documented in the literature [15,20,41].

Ferulic acid (FA) is an important substituent in arabinoxylan molecules [42,43,44,45]. Its presence influences the unique properties of these polysaccharides. SEC analysis with UV detection at 320 nm performed in this study allowed characterization of the relative FA content in the obtained arabinoxylan preparations as proportional to the surface area under the peak (Table 3) detected at 320 nm [15]. The content of FA in the tested water-soluble arabinoxylan preparations derived from whole-grain flours obtained from different rye varieties showed significant variation. The wide range of surface areas observed for samples with the highest and lowest ferulic acid content indicates up to a two-fold difference in the ferulic acid content in isolated AXs molecules. Statistical analysis showed that the ferulic acid content in AX molecules extracted from grain depended especially on the year of cultivation, and secondarily on cultivar and location of cultivation as well as their interactions. Higher FA levels were observed in AXs isolated from grain harvested in 2019 and from grain grown at the Osiny site. It is well established that environmental conditions and genotype significantly affect ferulic acid (FA) biosynthesis in rye grain. Stress conditions (such as dehydration) generally increase phenolic compounds, including FA, as a defense mechanism, while some farming practices (intensive vs. integrated) often have effects stronger than genotype [15,46]. The observed association between ferulic acid content and harvest year and growing location has not been reported previously. The highest FA content was found in rye flour obtained from the KWS Dolaro and Tur varieties, i.e., the varieties whose AXs had the lowest molar mass compared to the other flours analyzed (Table 3). Based on the analysis of wholemeal samples (Table 3) it was observed for the first time that the FA content was lower in high-molar-mass AX molecules while low-molar-mass AXs molecules were richer in this phenolic acid.

### 2.4. Structure of Arabinoxylans in Tested Rye Flour Samples

The uneven distribution of arabinoxylans within the rye kernel—and the consequent variation in the proportions of I-AX and S-AX fractions across different kernel parts—combined with the milling method, significantly influences the degree of arabinose substitution (A/X ratio) of specific arabinoxylan fractions in the resulting flour [1,38,47]. In rye flour, the A/X ratio typically ranges from 0.66 to 0.77, a finding supported by numerous studies [48,49]. By comparison, the A/X ratio in wheat flour is 0.50–0.70, indicating a higher degree of xylose chain substitution in rye AX [49]. In the present study (Table 4), the degree of xylose chain substitution in the I-AX_F_ fraction ranged from 0.73 to 0.85. Flour obtained from the Dańkowskie Skand rye cultivar exhibited the highest A/X ratio (0.81). The lowest degree of arabinoxylan chain substitution was observed in flour from the Dańkowskie Hadron cultivar (0.76). No significant effect of the cultivation year or location on the I-AX_F_ substitution degree was observed; the mean value in both cases was 0.79. The A/X ratio of I-AX_F_ was most strongly influenced by the genetic factor, as well as the X × Z and X × Y × Z interactions. In the analyzed flours, the mean degree of polymer chain substitution in the soluble arabinoxylan fraction was significantly lower than that in the A/X I-AX_F_ fraction, ranging from 0.62 to 0.74. Slight but significant differences in the A/X values of the S-AX_F_ fraction were observed depending on the year and cultivation location. Flour obtained from the KWS Dolaro rye cultivar exhibited the highest degree of A/X substitution, at 0.71. For the flours studied, the degree of substitution of the soluble arabinoxylan chain by arabinose residues was significantly influenced by each of the analyzed factors—genetic, environmental, and the interactions between them.

The molar mass of AX isolated from low-extraction rye flour was within the range of 1.27 × 10^6^–2.35 × 10^6^ g/mol (Table 4). The average molar mass of AX from flour was lower than that in the whole grain (Table 3). This observation shows that water-extractable AXs localized in the outer parts of the grain have significantly higher molar mass. Statistical analysis showed that molar mass of AX from rye flour depended on rye grain cultivar, year of cultivation and their interactions, whereas the effect of the cultivation location was not statistically significant. The relatively strong influence of cultivars on the molar mass of AXs was similar to that observed in whole grain, and similarly to the results presented in Table 3 AX from rye grain cultivars Dańkowskie and Piastowskie had higher molar mass compared to KWS Dolaro and Tur. Similarly to AX from whole grain, the molar mass of AX in rye flour obtained from grain cultivated in 2020 was slightly higher than that of AX from 2019.

The range of FA content, expressed as FA peak area (Table 4), observed on SEC/UV profiles was significantly lower than the range observed for AXs extracted from wholemeal. This means that AXs molecules from flour were significantly less substituted with FA compared to AX from wholemeal. The FA content, represented by the FA peak area in AXs extracted from rye flour, depended on year of cultivation, location and cultivar, however the influence of year of cultivation was most pronounced. It was found that in the case of AXs from rye flour obtained from grain cultivated in 2019 FA peak area was significantly lower than that in grain from 2020. This relationship was opposite to that observed for AXs from whole grain (Table 3) and could be explained by differences in the FA substitution of AXs molecules occurring in outer parts of rye grain. In 2019 whole grain contained AX molecules with a low degree of FA substitution and in 2020 the substitution of AXs in whole grain with FA was high. It was also found that in the case of AXs from rye flour obtained from grain cultivated in Osiny substitution of AXs by FA was significantly lower compared to grain cultivated in Grabów. This relationship also differed from that observed for AX from whole grain (Table 3) and similarly could be explained by differences in FA substitution in outer parts of rye grain. In Osiny the outer parts of rye grain contained AXs molecules with a low degree of FA substitution and in Grabów the substitution of outer parts of rye grain with FA was high. The influence of cultivar on FA substitution of AXs molecules extracted from flour was generally similar to AXs extracted from wholemeal. AXs molecules from flour obtained from rye grain of KWS Dolaro and Tur were more highly substituted with FA than those from Dańkowskie Granat, Dańkowskie Turkus, and Piastowskie. However, AX extracted from flour obtained from Dańkowskie Skand and Dańkowskie Hadron grain showed a high degree of FA substitution. It could be concluded that in the case of these cultivars AXs molecules occurring in inner parts of rye grain were highly substituted with FA.

## 3. Materials and Methods

### 3.1. Material

The research material consisted of grain from seven rye cultivars grown under organic farming conditions, harvested in 2019–2020 at two locations: Osiny and Grabów, as well as flour obtained from this grain with an extraction rate of 55%. Each cultivar was grown in three separate experimental plots at each location in each year. Grain from each plot was collected, milled, and analyzed separately, providing three independent experimental replicates per cultivar × location × year combination. The individual plot served as the experimental unit in the statistical analysis. Among the analyzed cultivars, two were hybrid cultivars (Tur and KWS Dolaro), while the remaining five were population cultivars: Dańkowskie Granat, Dańkowskie Hadron, Dańkowskie Skand, Dańkowskie Turkus, and Piastowskie. A detailed description of soil and weather conditions during the growing seasons is provided in Stępniewska et al. [50], whereas milling conditions and the milling balance are described in Stępniewska et al. [51].

### 3.2. Methods

#### 3.2.1. Determination of Total Arabinoxylan Content and Water-Extractable and Water-Unextractable Fractions

AXs content and its water-extractable (S-AX/WE-AX) and water-unextractable (I-AX/WUE-AX) fractions were determined using gas chromatography (GC) according to the method of Englyst and Cummings [52] for the analysis of nonstarch polysaccharides (NSP). In this procedure, the NSP content of each fraction was calculated as the sum of the five individual monomers arabinose, xylose, mannose, galactose, and glucose. After the enzymatic hydrolysis of starch, the samples were centrifuged and split into soluble (ethanol precipitates from supernatant) and insoluble (remaining pellet) fractions. Each of these fractions was hydrolyzed with 1 M sulfuric acid (100 °C, 2 h) to monosaccharides and converted to volatile alditol acetates. The alditol acetates were separated on a capillary quartz column Rtx-225 (0.53 mm × 30 m) using the Clarus 600 gas chromatograph (Perkin Elmer, Waltham, MA, USA) equipped with an autosampler, splitter injection port, and flame ionization detector. The carrier gas was He. Separation was performed at 225 °C, with injection and detection at 275 °C. For each fraction, the arabinoxylans content was calculated as the sum of the arabinose and xylose contents. The arabinose-to-xylose ratio (A/X) was also calculated.

#### 3.2.2. Extraction of Water-Extractable Rye Arabinoxylans and Analysis of Its Molar Mass and Ferulic Acid Content

Extraction and purification of extractable arabinoxylans were performed according to Shewry [53]. Briefly, 1 g of flour/wholemeal was treated with 5 mL of 50% ethanol for 15 min in a boiling water bath. After cooling and centrifugation, the pellet was suspended in 20 mL sodium acetate buffer (0.1 M). The suspension was incubated with thermostable α-amylase for 90 min in a boiling water bath. After cooling to room temperature amyloglucosidase was added and the sample was incubated at 60 °C overnight. After cooling and centrifugation, 15 mL of the supernatant was transferred to a new tube, and 450 μL of 0.5 N sodium phosphate was added. The mixture was then treated with 1 mg of pancreatin and 100 μL of lichenase and incubated at 40 °C for 2 h. After this period 135 mL of 99% ethanol was added and the sample was centrifuged. After centrifugation the pellet was resuspended in 2 mL of 0.1 M sodium nitrate in a boiling water bath for 10 min, centrifuged, and the supernatant (extracted water-extractable arabinoxylans) was filtered through a 0.45 um filter.

The molar mass of water-extractable arabinoxylan was determined by SEC. 100 μL of extracted water soluble arabinoxylan, dissolved in SEC mobile phase (100 mM NaNO_3_) was injected into the SEC system equipped with a combination of OHpak (Shodex, Tokyo, Japan) columns (SB-G, SB-806HQ, SB-804HQ, and SB-802HQ). The carbohydrates (AX) were detected with RI detection while UV-absorbing compounds associated with feruloylated AX were monitored at 320 nm using a UV detector as described by [44]. The area of the UV peak at 320 nm was used as a relative parameter for comparison among samples. Since no purified feruloylated arabinoxylan standard was available for calibration, the 320 nm UV peak area was not used for absolute quantification of ferulic acid and is therefore reported as a relative ferulic-acid-associated UV response. The separation was done at a flow rate of 0.6 mL/min and a column temperature of 60 °C. The calibration of the SEC system was done with pullulan standards and arabinose (Sigma–Aldrich, Warsaw, Poland). The apparent average molar mass M_w_, calculated relative to pullulan standards as well as ferulic acid peak area were calculated using the software programs Eurochrom ver. 3.05 (Knauer, Berlin, Germany) and Clarity ver. 4.0.1.700 (DataApex, Praga, Czech Republic).

#### 3.2.3. Statistical Analysis

All measurements were performed on three independent experimental replicates per cultivar × location × year combination, with each replicate consisting of material from a separate plot. A three-way analysis of variance (ANOVA) was performed to assess the effects of harvest year (two levels), location (two levels), cultivar (seven levels), and their interactions on the analyzed traits. Post-hoc comparisons were performed using Tukey’s test. Statistical significance was determined at the levels of α = 0.01 and α = 0.05. All statistical analyses were conducted using R software (version 4.2.1).

## 4. Conclusions

The study demonstrated that the properties of arabinoxylans in rye grain and low-extraction flours obtained from seven rye cultivars (hybrid and population cultivars) grown under organic farming conditions were significantly affected by genotype, environmental factors, and their interactions. These factors affected arabinoxylan content, molar mass, degree of arabinose substitution, and ferulic acid content. Rye grain exhibited a higher total AXs content but a lower proportion of the water-soluble fraction of AXs, compared with flour. The degree of arabinose substitution varied depending on the arabinoxylan fraction and the analyzed material. Water-unextractable AXs and AXs extracted from flour showed a higher degree of substitution than the water-extractable fraction of AX and AX isolated from the whole grain. The molar mass of water-extractable AX ranged from 1.53 × 10^6^ to 2.79 × 10^6^ g/mol in grain and was lower in flour (1.27 × 10^6^ to 2.35 × 10^6^ g/mol), indicating differences in the molecular characteristics of AXs between grain and low-extraction flour. The observed relationships between AXs molar mass and ferulic acid content suggest differences in the degree of ferulic acid substitution among AX of different molecular sizes. Overall, the results demonstrate that genotype and environmental conditions influence the structural characteristics of AXs in rye grown under organic farming conditions. These findings may be relevant to rye breeding and cereal processing aimed at obtaining flours with specific AXs characteristics and technological functionality.

## Figures and Tables

**Table 1:** The content (% d.m.) of arabinoxylans in the tested rye grain samples.

Effects	Rye Grain			
	T-AX	S-AX	I-AX	%S-AX
Range	7.18–9.12	2.08–2.76	4.56–6.50	24–37
Harvest year				
2019	7.56 ± 0.27 ^b^	2.47 ± 0.12 ^a^	5.09 ± 0.31 ^b^	33 ± 2 ^a^
2020	8.22 ± 0.43 ^a^	2.37 ± 0.17 ^b^	5.85 ± 0.32 ^a^	28 ± 2 ^b^
Location				
Osiny	7.94 ± 0.54 ^a^	2.45 ± 0.14 ^a^	5.49 ± 0.12	31 ± 2 ^a^
Grabów	7.84 ± 0.44 ^b^	2.38 ± 0.16 ^b^	5.47 ± 0.12	29 ± 3 ^b^
Cultivar				
Tur	7.47 ± 0.21 ^c^	2.41 ± 0.23 ^b^	5.05 ± 0.44 ^b^	33 ± 4 ^a^
KWS Dolaro	7.95 ± 0.15 ^ab^	2.35 ± 0.06 ^b^	5.61 ± 0.18 ^a^	29 ± 1 ^b^
Dańkowskie Granat	8.02 ± 0.62 ^ab^	2.50 ± 0.08 ^a^	5.52 ± 0.66 ^a^	31 ± 3 ^a^
Dańkowskie Hadron	7.93 ± 0.38 ^ab^	2.38 ± 0.16 ^b^	5.57 ± 0.53 ^a^	30 ± 3 ^b^
Dańkowskie Skand	8.04 ± 0.80 ^a^	2.51 ± 0.20 ^a^	5.52 ± 0.68 ^a^	32 ± 2 ^a^
Dańkowskie Turkus	7.95 ± 0.52 ^ab^	2.40 ± 0.10 ^b^	5.55 ± 0.53 ^a^	30 ± 2 ^b^
Piastowskie	7.89 ± 0.34 ^b^	2.36 ± 0.14 ^b^	5.54 ± 0.29 ^a^	30 ± 1 ^b^
ANOVA				
Factor	F			
Harvest year (X)	1032.3 **	72.9 **	980.5 **	495.1 **
Location (Y)	20.5 **	38.0 **	0.7 ^ns^	9.6 **
Cultivar (Z)	50.4 **	17.8 **	34.1 **	19.7 **
X × Y	10.7 **	63.9 **	1.2 ^ns^	26.8 **
X × Z	79.0 **	34.4 **	37.7 **	18.1 **
Y × Z	20.2 **	10.4 **	19.3 **	12.7 **
X × Y × Z	36.4 **	16.5 **	20.3 **	9.9 **

ns—not significant; ** significant difference at *p* ≤ 0.01; a–c—homogeneous groups (*p* < 0.05). Detailed explanations: T-AX—total arabinoxylans content in grain; S-AX—water-extractable arabinoxylans content in grain; I-AX—water-unextractable arabinoxylans content in grain; %S-AX—the share of S-AX in the T-AX in grain.

**Table 2:** The content (% d.m.) of arabinoxylans in the rye flour obtained from tested rye grain samples.

Effects	Rye Flour			
	T-AX_F_	S-AX_F_	I-AX_F_	%S-AX_F_
Range	2.87–4.17	1.74–2.44	0.85–2.16	47–70
Harvest year				
2019	3.18 ± 0.24 ^b^	2.07 ± 0.19 ^a^	1.11 ± 0.13 ^a^	65 ± 7 ^a^
2020	3.70 ± 0.29 ^a^	1.95 ± 0.09 ^b^	1.75 ± 0.25 ^b^	53 ± 7 ^b^
Location				
Osiny	3.31 ± 0.33 ^a^	2.01 ± 0.11	1.30 ± 0.34 ^b^	61 ± 7 ^a^
Grabów	3.58 ± 0.36 ^b^	2.00 ± 0.20	1.57 ± 0.38 ^a^	56 ± 7 ^b^
Cultivar				
Tur	3.53 ± 0.63 ^a^	1.98 ± 0.16 ^b^	1.55 ± 0.51 ^a^	57 ± 7 ^e^
KWS Dolaro	3.32 ± 0.37 ^c^	1.90 ± 0.11 ^c^	1.42 ± 0.41 ^b^	58 ± 8 ^d^
Dańkowskie Granat	3.44 ± 0.21 ^b^	2.09 ± 0.22 ^a^	1.35 ± 0.27 ^c^	61 ± 7 ^a^
Dańkowskie Hadron	3.59 ± 0.27 ^a^	2.08 ± 0.14 ^a^	1.51 ± 0.32 ^a^	58 ± 6 ^d^
Dańkowskie Skand	3.42 ± 0.28 ^b^	2.04 ± 0.12 ^ab^	1.38 ± 0.32 ^bc^	60 ± 6 ^b^
Dańkowskie Turkus	3.40 ± 0.34 ^bc^	1.98 ± 0.19 ^b^	1.41 ± 0.39 ^b^	59 ± 8 ^c^
Piastowskie	3.41 ± 0.40 ^b^	2.00 ± 0.14 ^b^	1.42 ± 0.48 ^b^	59 ± 9 ^c^
ANOVA				
Factor	F			
Harvest year (X)	1131.1 **	117.6 **	4934.2 **	16,411.0 **
Location (Y)	299.9 **	0.2 ^ns^	877.6 **	2397.0 **
Cultivar (Z)	19.5 **	18.2 **	35.2 **	107.0 **
X × Y	10.9 **	3.4 ^ns^	61.9 **	24.0 **
X × Z	72.0 **	40.4 **	50.1 **	44.0 **
Y × Z	25.3 **	17.6 **	24.4 **	65.0 **
X × Y × Z	18.7 **	20.7 **	36.5 **	174.0 **

ns—not significant; ** significant difference at *p* ≤ 0.01; a–e—homogeneous groups (*p* < 0.05). Detailed explanations: T-AX_F_—total arabinoxylans content in flour; S-AX_F_—water-extractable arabinoxylans content in flour; I-AX_F_—water-unextractable arabinoxylans content in flour; %S-AX_F_—the share of S-AX in the T-AX in flour.

**Table 3:** Structural characteristics of arabinoxylans in tested rye grain.

Effects	Rye Grain			
	A/X I-AX	A/X S-AX	M_W_ × 10^6^ (g/mol)	FA Peak Area (a.u.)
Range	0.59–0.77	0.59–0.68	1.53–2.79	3157–8012
Harvest year				
2019	0.67 ± 0.03 ^a^	0.63 ± 0.02 ^a^	2.01 ± 0.24 ^b^	6615 ± 861 ^a^
2020	0.67 ± 0.04 ^a^	0.64 ± 0.02 ^a^	2.15 ± 0.32 ^a^	5080 ± 919 ^b^
Location				
Osiny	0.68 ± 0.04 ^a^	0.64 ± 0.02 ^a^	2.02 ± 0.24 ^b^	5987 ± 929 ^a^
Grabów	0.67 ± 0.04 ^a^	0.63 ± 0.02 ^a^	2.12 ± 0.31 ^a^	5710 ± 1381 ^b^
Cultivar				
Tur	0.67 ± 0.03 ^cd^	0.63 ± 0.02 ^ab^	1.70 ± 0.18 ^f^	6373 ± 1567 ^a^
KWS Dolaro	0.71 ± 0.03 ^a^	0.65 ± 0.02 ^a^	1.90 ± 0.05 ^e^	6446 ± 89 ^a^
Dańkowskie Granat	0.64 ± 0.03 ^f^	0.64 ± 0.03 ^ab^	2.01 ± 0.07 ^d^	5521 ± 1929 ^d^
Dańkowskie Hadron	0.68 ± 0.05 ^b^	0.65 ± 0.02 ^a^	2.39 ± 0.17 ^a^	5815 ± 576 ^c^
Dańkowskie Skand	0.65 ± 0.04 ^ef^	0.62 ± 0.01 ^b^	2.15 ± 0.28 ^c^	6049 ± 734 ^b^
Dańkowskie Turkus	0.68 ± 0.03 ^b^	0.63 ± 0.01 ^ab^	2.23 ± 0.29 ^b^	5430 ± 724 ^d^
Piastowskie	0.67 ± 0.03 ^cd^	0.64 ± 0.02 ^ab^	2.18 ± 0.19 ^bc^	5302 ± 1351 ^e^
ANOVA				
Factor	F			
Harvest year (X)	0.1 ^ns^	2.13 ^ns^	91.34 *	8160 *
Location (Y)	3.2 ^ns^	1.69 ^ns^	22.14 *	265 *
Cultivar (Z)	14.9 *	3.28 **	148.88 *	413 *
X × Y	14.8 *	3.65 ^ns^	80.38 *	353 *
X × Z	11.7 *	2.17 ^ns^	50.50 *	955 *
Y × Z	3.1 *	0.60 ^ns^	11.50 *	158 *
X × Y × Z	19.1 *	1.12 ^ns^	12.02 *	130 *

ns—not significant; ** significant difference at *p* ≤ 0.01; * significant difference at *p* ≤ 0.05; a–f—homogeneous groups (*p* < 0.05). Detailed explanations: A/X I-AX—degree of branching of water-water-unextractable arabinoxylans in grain; A/X S-AX—degree of branching of water-extractable soluble arabinoxylans content in grain; M_W_—molecular mass of S-AX in grain; FA peak area—the value proportional to the content of ferulic acid in arabinoxylans extracted from grain.

**Table 4:** Structural characteristics of arabinoxylans of tested rye flour.

Effects	Rye Flour			
	A/X I-AX_F_	A/X S-AX_F_	M_Wflour_ × 10^6^ (g/mol)	FA_F_ Peak Area (a.u.)
Range	0.73–0.85	0.62–0.74	1.27–2.35	1508–3812
	Harvest year			
2019	0.79 ± 0.03 ^a^	0.68 ± 0.03 ^b^	1.93 ± 0.17 ^b^	2167 ± 391 ^b^
2020	0.79 ± 0.03 ^a^	0.70 ± 0.03 ^a^	1.97 ± 0.33 ^a^	2675 ± 427 ^a^
Location				
Osiny	0.79 ± 0.03 ^a^	0.68 ± 0.02 ^b^	1.94 ± 0.28 ^a^	2328 ± 351 ^b^
Grabów	0.79 ± 0.03 ^a^	0.70 ± 0.02 ^a^	1.95 ± 0.25 ^a^	2515 ± 573 ^a^
Cultivar				
Tur	0.79 ± 0.03 ^abc^	0.68 ± 0.02 ^ab^	1.72 ± 0.26 ^f^	2496 ± 514 ^b^
KWS Dolaro	0.79 ± 0.02 ^ab^	0.71 ± 0.03 ^a^	1.85 ± 0.38 ^e^	2787 ± 620 ^a^
Dańkowskie Granat	0.80 ± 0.04 ^ab^	0.67 ± 0.04 ^b^	2.02 ± 0.19 ^b^	2247 ± 331 ^c^
Dańkowskie Hadron	0.76 ± 0.02 ^c^	0.69 ± 0.03 ^ab^	1.96 ± 0.04 ^cd^	2508 ± 377 ^b^
Dańkowskie Skand	0.81 ± 0.03 ^a^	0.68 ± 0.03 ^b^	2.18 ± 0.10 ^a^	2753 ± 217 ^a^
Dańkowskie Turkus	0.78 ± 0.02 ^bc^	0.69 ± 0.04 ^ab^	2.00 ± 0.31 ^bc^	2027 ± 477 ^e^
Piastowskie	0.78 ± 0.02 ^bc^	0.68 ± 0.03 ^b^	1.92 ± 0.23 ^d^	2133 ± 189 ^d^
ANOVA				
Factor	F			
Harvest year (X)	0.9 ^ns^	14.3 *	23.3 *	2095 *
Location (Y)	0.4 ^ns^	9.8 *	2.5 ^ns^	285 *
Cultivar (Z)	6.9 *	3.6 *	164.2 *	406 *
X × Y	6.1 **	21.2 *	51.6 *	94 *
X × Z	4.8 *	8.1 *	67.0 *	138 *
Y × Z	2.7 **	3.7 *	98.6 *	128 *
X × Y × Z	7.1 *	4.9 *	295.0 *	133 *

ns—not significant; ** significant difference at *p* ≤ 0.01; * significant difference at *p* ≤ 0.05; a–f—homogeneous groups (*p* < 0.05). Detailed explanations: A/X I-AX_F_—degree of branching of water-unextractable arabinoxylans in flour; A/X S-AX_F_—degree of branching of water-extractable arabinoxylans in flour; M_Wflour_—molecular mass of S-AX in flour; FA peak area—the value is proportional to the content of ferulic acid in arabinoxylans extracted from flour.

## Data Availability

The original contributions presented in the study are included in the article, further inquiries can be directed to the corresponding authors.

## Abbreviations

The following abbreviations are used in this manuscript:

AXs	Arabinoxylans
NSP	Nonstarch polysaccharides
T-AX	Total arabinoxylans content in grain
S-AX	Water soluble arabinoxylans content in grain
I-AX	Insoluble arabinoxylans content in grain
%S-AX	The share of S-AX in the T-AX in grain
T-AX_F_	Total arabinoxylans content in flour
S-AX_F_	Water soluble arabinoxylans content in flour
I-AX_F_	Insoluble arabinoxylans content in flour
%S-AX_F_	The share of S-AX in the T-AX in flour
A/X ratio	Degree of substitution of the xylan backbone with arabinose residues
A/X I-AX	Degree of branching of insoluble arabinoxylans in grain
A/X S-AX	Degree of branching of water soluble arabinoxylans in grain
A/X I-AX_F_	Degree of branching of insoluble arabinoxylans in flour
A/X S-AX_F_	Degree of branching of water soluble arabinoxylans in flour
